# Plasmalogens and Chronic Inflammatory Diseases

**DOI:** 10.3389/fphys.2021.730829

**Published:** 2021-10-21

**Authors:** José Carlos Bozelli, Sayed Azher, Richard M. Epand

**Affiliations:** Department of Biochemistry and Biomedical Sciences, Health Sciences Centre, McMaster University, Hamilton, ON, Canada

**Keywords:** plasmalogen, polyunsaturated fatty acids, oxidative stress, inflammation, aging, degenerative disorders, metabolic disorders, plasmalogen replacement therapy

## Abstract

It is becoming widely acknowledged that lipids play key roles in cellular function, regulating a variety of biological processes. Lately, a subclass of glycerophospholipids, namely plasmalogens, has received increased attention due to their association with several degenerative and metabolic disorders as well as aging. All these pathophysiological conditions involve chronic inflammatory processes, which have been linked with decreased levels of plasmalogens. Currently, there is a lack of full understanding of the molecular mechanisms governing the association of plasmalogens with inflammation. However, it has been shown that in inflammatory processes, plasmalogens could trigger either an anti- or pro-inflammation response. While the anti-inflammatory response seems to be linked to the entire plasmalogen molecule, its pro-inflammatory response seems to be associated with plasmalogen hydrolysis, *i.e*., the release of arachidonic acid, which, in turn, serves as a precursor to produce pro-inflammatory lipid mediators. Moreover, as plasmalogens comprise a large fraction of the total lipids in humans, changes in their levels have been shown to change membrane properties and, therefore, signaling pathways involved in the inflammatory cascade. Restoring plasmalogen levels by use of plasmalogen replacement therapy has been shown to be a successful anti-inflammatory strategy as well as ameliorating several pathological hallmarks of these diseases. The purpose of this review is to highlight the emerging role of plasmalogens in chronic inflammatory disorders as well as the promising role of plasmalogen replacement therapy in the treatment of these pathologies.

## Plasmalogens

Plasmalogens are among the most common glycerophospholipids. These lipids have a broad phylogenetic distribution, being found in many biological membranes (bacteria, protozoa, invertebrates, and mammals) (Braverman and Moser, 2012). In most mammalian membranes they comprise approximately 15 to 20% of total membrane phospholipids (Han et al., 2001; Braverman and Moser, 2012). Thus, plasmalogens are among the major lipid components of these membranes. Despite their abundance, plasmalogens had received relatively little attention compared with many other lipid classes over the years. However, lately, plasmalogens have begun to attract more attention due to their association with several degenerative and metabolic disorders as well as aging. In the present review, the focus will be the role of plasmalogens in mammalian inflammatory processes.

### Distribution Among Species

Plasmalogens have been found in bacteria, protozoa, invertebrates, and mammals. However, they are not found in plants and, likely, are not present in fungi (Horrocks and Sharma, 1982; Felde and Spiteller, 1994). Among bacteria, they are found in anaerobic bacteria but not in aerobic or facultative aerobic bacteria. The chemical structure of bacterial plasmalogens differs from that of mammals (see below).

### Distribution Within the Organism

Plasmalogens are widely distributed within the mammalian organism. They are found in a variety of organs, cells, and other lipid-rich structures such as the myelin sheath and lipoproteins (Table 1). The highest amount of plasmalogen is found in the brain, while the liver has the lowest amount of plasmalogens (Koch et al., 2020). Choline plasmalogens (also called plasmenylcholine, PC-Pls) are highly enriched in the heart and smooth muscle, while all other organs are enriched with ethanolamine plasmalogens (also called plasmenylethanolamine, PE-Pls).

**TABLE 1:** Variation of plasmalogen content among different mammalian organs/structures.

**Organ/Cell/Lipid-rich structure**	**Total Plasmalogen (% of total phospholipid)**	**PE-Pls (% of total phospholipid)**	**PC-Pls (% of total phospholipid)**	**References**
LDL	10	4 (60% of total PE)	4 (4% of total PC)	Bräutigam et al., 1996; Ikuta et al., 2019
HDL	10	5 (55% of total PE)	4 (5% of total PC)	
Brain gray matter	10	10 (49% of total PE)	N.D.	O’brien et al., 1965
Brain white matter	12	12 (86% of total PE)	N.D.	
Myelin	12	12 (90% of total PE)	N.D.	
CNS cell culture	9	8 (49% of total PE)	1 (3% of total PC)	Fitzner et al., 2020
Heart	32	15 (54% of total PE)	17 (42% of total PC)	Hughes and Frais, 1967
Macrophages	15	13 (61% of total PE)	2 (6% of total PC)	Sugiura et al., 1983
Spermatozoa	12	9 (30% of total PE)	3 (9% of total PC)	Poulos and White, 1973

*Total = PE-Pls + PC-Pls (other minor plasmalogens not considered).*

*N.D. = non-detected or trace amounts.*

*CNS = Central nervous system*

*CNS cell culture is the average of astrocytes, microglia, neurons, and oligodendrocytes.*

### Chemical Structure

#### The Enyl-Ether Linkage

**FIGURE 1:**
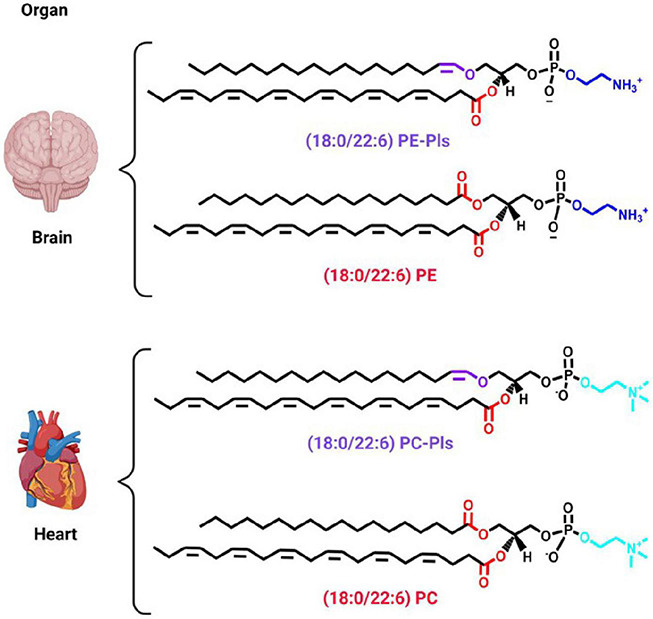
Predominant plasmalogen species in different organs. A cartoon showing two organs, *i.e.*, brain and heart, to exemplify the chemical structure of the predominant plasmalogen species in those organs. Plasmenylethanolamine [18:0/22:6 PE-Pls, 1-(1Z-octadecenyl)-2-docosahexaenoyl-*sn*-glycero-3-phosphoethanolamine] is the predominant plasmalogen species in the brain and plasmenylcholine [18:0/22:6 PC-Pls, 1-(1Z-octadecenyl)-2-docosahexaenoyl-*sn*-glycero-3-phosphoethanolamine] the one in the heart (Koch et al., 2020). The chemical structure of their diacyl counterparts is also shown for comparison. Ethanolamine and choline headgroups are colored in dark and light blue, respectively. The ester and vinyl-ether bonds are colored in red and purple, respectively. The remaining chemical structures are in black. Schematic representations were generated using Biorender (©BioRender - biorender.com).

Plasmalogens are a subclass of glycerophospholipids. As such they have a similar chemical structure to diacyl glycerophospholipids. The difference lies in the linkage at the *sn*-1 position of the glycerol moiety (Figure 1). While diacyl glycerophospholipids bear an ester bond, plasmalogens present an ether bond. The ether bond links an alkyl chain to the glycerol backbone. Plasmalogens, however, differ from other ether lipids in having a double bond adjacent to the ether linkage, thus making them enyl-ether (vinyl-ether) lipids (Figure 1). The enyl-ether linkage imparts differences in the physical, chemical, and biological properties of plasmalogens in comparison to their diacyl counterparts (Nagan and Zoeller, 2001; Braverman and Moser, 2012). From the chemical point of view, the enyl-ether bond is (i) more hydrophobic, (ii) more acid labile, (iii) more oxidation labile, as well as (iv) less involved in hydrogen bonding than their diacyl counterparts (Gorgas et al., 2006).

#### The Alkyl Chain

The enyl-ether bond links an alkyl chain at the *sn*-1 position of the glycerol moiety in plasmalogens (Figure 1). The most abundant alkyl chains in plasmalogens, especially in mammals, are 16 and 18 carbon atoms long (Koivuniemi, 2017; Koch et al., 2020). These chains are, usually, saturated, or monounsaturated. In bacteria, there are also chains with an odd number of carbon atoms, as well as unsaturated alkyl chains at the *sn*-1 position (Nagan and Zoeller, 2001; Řezanka et al., 2011; Farooqui and Horrocks, 2012).

#### The Acyl Chain

As with their diacyl glycerophospholipid counterparts, plasmalogens bear an acyl chain at the *sn*-2 position of the glycerol backbone attached via an ester bond (Figure 1). The predominant acyl chains are 20-22 carbon atoms long and are polyunsaturated (Gorgas et al., 2006; Koivuniemi, 2017). The two most common acyl chains in this position are the polyunsaturated fatty acids (PUFA) docosahexaenoic acid (DHA, 22:6, an ω-3 fatty acid) and arachidonic acid (AA, 20:4, an ω-6 fatty acid) (Fuchs, 2015; Koch et al., 2020). Plasmalogens from the brain and heart are enriched with DHA, while the colon and spleen are enriched with AA (Koch et al., 2020). In mice, plasmalogens from the kidney are enriched with DHA in males and AA in females (Koch et al., 2020). At the *sn*-2 position, bacterial plasmalogens contains branched and saturated acyl chains (Řezanka et al., 2011).

#### The Headgroup

The two most common alcohols that are linked to the *sn*-3 position of the glycerol moiety of plasmalogens are choline and ethanolamine. In addition, small amounts of plasmalogens with serine and inositol headgroups have been detected, but only as minor components (Fuchs, 2015). In bacteria, in addition to ethanolamine, serine and glycerol are also found as major components (Řezanka et al., 2011, 2012). Additionally, the plasmalogen form of cardiolipin has also been identified in bacteria (Johnston and Goldfine, 1994).

### Physical Properties

Plasmalogens have many effects on the physical properties of biological membranes. For instance, they rigidify membranes, lower the fluidity and stabilize the formation of membrane domains as well as stabilize negatively curved surfaces. All of which have been suggested to contribute to their cellular function.

The enyl-ether linkage has substantial effects on the conformation and dynamics of plasmalogens (Koivuniemi, 2017). For instance, the enyl-ether bond changes the conformation of the lipid headgroup. In PC-Pls this change is reflected in the choline headgroup oriented more toward the water than the bilayer-water interface when compared to its diacyl counterpart (Han and Gross, 1990; Koivuniemi, 2017). The enyl-ether bond also changes the conformation and dynamics of the acyl chain at the *sn*-2 position of the glycerol. It has been shown that this acyl chain in plasmalogens has greater motional freedom compared with its diacyl counterpart. However, in PC-Pls this increased motional freedom is lost at lower temperatures or in the presence of cholesterol (Malthaner et al., 1987).

The enyl-ether bond causes closer packing of the proximal regions of the alkyl-acyl chains in PC-Pls in comparison to its diacyl counterpart (Han and Gross, 1990). Monolayer studies have shown a lower molecular area for PC-Pls compared with the diacyl or alkyl/acyl (plasmanyl, an ether lipid without the enyl-ether double bond) counterparts (Smaby et al., 1983). The differences were less pronounced for PE-Pls. However, the reverse order was found in molecular dynamic studies (Pietiläinen et al., 2011; Rog and Koivuniemi, 2016). A possible cause for this disagreement is that the monolayer studies were done using plasmalogens with an arachidonoyl group (20:4) at the *sn*-2 position, but the molecular dynamics studies used an oleoyl (18:1) acyl chain. The presence of cholesterol reduced the difference in lateral pressure between the diacyl and enyl-ether lipids. Molecular dynamics showed a high compression at the glycerol backbone for the plasmalogens, resulting in a reduced cross-sectional area of the headgroup (Janmey and Kinnunen, 2006). This results in a slightly thicker bilayer and lower area per lipid molecule.

Simulations also show an increased orientational ordering along the bilayer normal of both the *sn*-1 and *sn*-2 chains in PC-Pls (Koivuniemi, 2017). These chains are more ordered resulting in a more rigid bilayer. This is compatible with plasmalogens being sequestered in liquid-ordered domains in membranes. Indeed, there is evidence that plasmalogens are not distributed uniformly along the plane of the membrane. For instance, they have been shown to be highly enriched in raft-like domains (Pike et al., 2002). Lipidomic analysis has suggested that plasmalogens increase the stability of lipid domains in rat synaptosome membranes (Tulodziecka et al., 2016). Plasmalogens also affect membrane fluidity. Fluorescent probe studies using membranes of *Megasphaera elsdenii* showed that membranes depleted of plasmalogens have a lower order parameter than control membranes (Kaufman et al., 1990). It has been shown that plasmalogen-rich nematode exosomes had increased rigidity compared with murine cells (Simbari et al., 2016).

The enyl-ether linkage is also less compatible with forming hydrogen bonds with water, making the membrane surface more hydrophobic, contributing to the tendency to form inverted phases. This agrees with the studies of Lohner and coworkers showing a lower bilayer to hexagonal phase transition temperature of plasmalogens (Lohner et al., 1984, 1991; Lohner, 1996). Plasmalogens also are enriched in membrane regions with high curvature, such as coated pits, the endoplasmic reticulum (ER), and Golgi cisterna (Thai et al., 2001).

While a considerable understanding of plasmalogen properties in model systems have been gathered, there are certain factors that have not been completely studied. One aspect is the dependence of the physical properties on specific molecular species of plasmalogens. As is described above there are many molecular species of plasmalogens. In many cases the effects of PC-Pls have been distinguished from those of PE-Pls, but in general, the sensitivity of the physical property to the nature of the acyl chain at the *sn*-2 position has not been studied. In addition, most of the studies have been done *in vitro/in silico* using pure plasmalogens or simple lipid mixtures with only two or at most three lipid components, compared with the complex lipid mixtures found in biological membranes. Furthermore, biological membranes have an asymmetric transbilayer distribution of lipids, which is known to affect membrane properties and their interaction with proteins (Bozelli et al., 2020a). However, most model system studies used symmetrical bilayers that are easier to prepare. Finally, biological membranes have a significant fraction of proteins that can facilitate the formation of membrane domains or can themselves affect membrane physical properties in a manner that would be absent in a model system. Despite these caveats, model system studies have clearly shown differences in the effect of plasmalogens on the physical properties of membranes and how their behavior differs from those of diacyl-lipids or plasmanyl lipids. Changes in membrane physical properties that are specific to plasmalogens must be considered as a possible cause of changes observed in biological membranes resulting from the presence of plasmalogens.

### Biological Properties

These manifestations of the effects of plasmalogens on membrane physical properties have been proposed to play a role in a variety of biological functions. Because of the chemical lability of the enyl-ether bond, plasmalogens are suggested to be protective agents against oxidation, acting as scavengers of radicals, such as reactive oxygen species (ROS) and reactive nitrogen species (RNS) (Reiss et al., 1997; Hahnel et al., 1999a; Zoeller et al., 2002). The ability to scavenger ROS/RNS is ascribed to the oxidation-labile enyl-ether bond. *In vitro* plasmalogens decrease the oxidative degradation of PUFA with an efficacy like vitamin E (a mitochondrial antioxidant used to prevent lipid peroxidation). Plasmalogens terminate lipid peroxidation since the products of plasmalogen oxidation are unable to further propagate oxidative reactions (Maulik et al., 1994; Sindelar et al., 1999; Khan et al., 2008; Dott et al., 2014). *In cellula*, plasmalogens increase the resistance of cells to oxidative stress (Zoeller et al., 2002). In addition, in brain white matter from cerebral adrenoleukodystrophy patients, plasmalogen levels are inversely correlated with ROS levels, *i.e.*, increased ROS leads to a decrease in plasmalogen (Khan et al., 2008). Likewise, administering plasmalogen precursors, which increased plasmalogen levels, to a rat model of reperfusion injury, reduced lipid peroxidation (Maulik et al., 1994). Furthermore, increasing plasmalogen levels protects human endothelial cells during hypoxia (Zoeller et al., 2002).

The formation and properties of lipid domains is under active investigation (Bozelli and Epand, 2018). Lipid domains are thicker than the surrounding membrane and they are in the liquid-ordered state that would favor the presence of plasmalogens. It is generally agreed, however, that these domains, in addition to plasmalogens, are also enriched in cholesterol and sphingomyelin and that these domains are of small size and are transient. There is also considerable evidence that lipid domains play an important role in many signal transduction pathways (Bozelli and Epand, 2018). Signal transduction can also occur due to the production of plasmalogen-derived lipid messengers, such as AA. The finding that lipid domains also have a high content of plasmalogens suggests a mechanism for their role in inflammation (Brites et al., 2004). Thus, in addition to the direct effects of plasmalogens on membrane physical properties, it is also likely that plasmalogens have a role in signal transduction (Dorninger et al., 2020).

Negative curvature lipids, particularly PE-Pls, increase the rate of fusion and stimulate extracellular and intracellular vesicle trafficking, particularly for synaptic vesicles (Glaser and Gross, 1994, 1995). There is also evidence that PE-Pls plays a role in the fusion of enveloped viruses with cell membranes (van Meer et al., 2008). This is the case for cytomegalovirus and influenza virus (Liu et al., 2011; Gerl and Sampaio, 2012). The membranes of some parasites are also enriched in plasmalogens (Brouwers et al., 1998; Villas Bôas et al., 1999; Simbari et al., 2016). Exosomes of parasites also contain high levels of plasmalogens in their membranes as do extracellular vesicles from platelets (Pienimaeki-Roemer et al., 2013). Plasmalogens in these vesicles may increase the rate of membrane fusion, however, the sequestration of plasmalogens in lipid domains may alter certain signaling pathways. Feeding PC-3 cells, a metabolic precursor of plasmalogens, hexadecyl-glycerol, causes a large increase in the release of exosomes (Phuyal et al., 2015). Plasmalogens cause the number of caveolae to be reduced and their size becomes smaller, and this lipid also affects axonal sorting and myelin formation (Gorgas et al., 2006; da Silva et al., 2014).

The small change in the chemical functional group from an ester to an enyl-ether, found in plasmalogens, has a dramatic impact on the effect of plasmalogens on membrane physical properties. Hence, both the chemistry, and physical properties of plasmalogens contribute to their role in a variety of cell biology phenomena.

## Metabolism of Plasmalogens

### Biosynthesis

**FIGURE 2:**
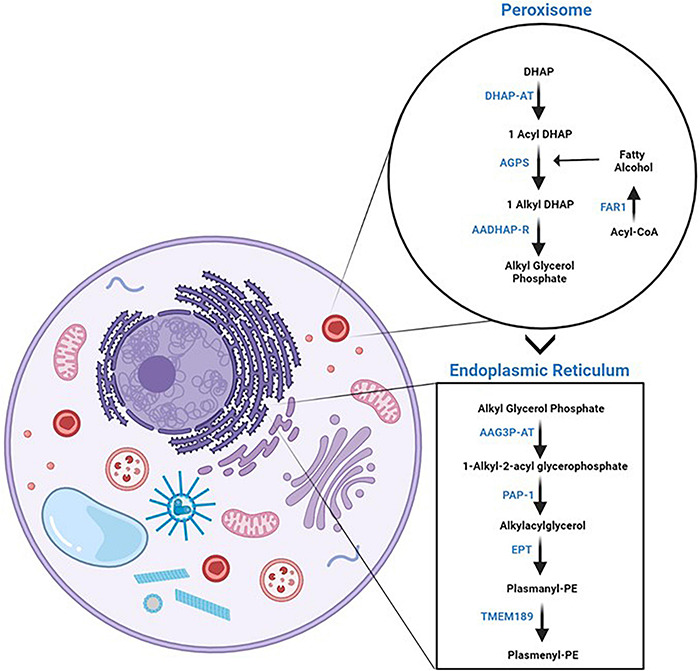
Plasmalogen *de novo* biosynthesis. On the left panel, a cartoon of a cell with a focus on the organelles where plasmalogen *de novo* biosynthesis takes place, *i.e.*, peroxisomes and endoplasmic reticulum. On the right panel, the enzymatic reactions of plasmalogen *de novo* biosynthetic pathway are separated by the organelles where they take place. The reader is referred to the text for the names of the enzymes. Schematic representations were generated using Biorender (©BioRender - biorender.com).

The *de novo* biosynthesis pathway of plasmalogens comprises enzymatic reactions that take place on both peroxisomes and ER (Figure 2) (Nagan and Zoeller, 2001; Wanders, 2014). The biosynthesis of PE-Pls is the best characterized one. Most evidence suggests that PC-Pls is formed from PE-Pls via headgroup transfer and/or remodeling and, therefore, both lipids have common biosynthetic pathways. PE-Pls biosynthesis is initiated in the peroxisome where dihydroxyacetone phosphate (DHAP) is esterified with acyl-CoA by a matrix peroxisomal enzyme, dihydroxyacetone phosphate acyltransferase (DHAP-AT) (Nagan and Zoeller, 2001; Wanders, 2014). The next enzyme in the pathway for the biosynthesis of PE-Pls, namely alkyl-DHAP synthase (AGPS), catalyzes the replacement of the fatty acid with a fatty alcohol attached to the *sn*-1 position via an ether bond. DHAP-AT and AGPS form a heterotrimeric complex, which is believed to facilitate substrate channeling. Both these enzymes are imported into the peroxisomal matrix via peptide signals in their sequences, which depend on transmembrane peroxisomal transporter pathways. The fatty alcohol is provided by a fatty acyl-CoA reductase, Far1, which is anchored to the cytoplasmic face of the peroxisomal membrane. The third reaction is catalyzed by an enzyme found at both peroxisome and ER and is a common point between the biosynthesis of both plasmalogens and diacyl phospholipids. This enzyme, acyl/alkyl-DHAP reductase (AADHAP-R) forms 1-alkyl-2-lyso-*sn*-glycero-3-phosphate by reducing the ketone at *sn*-2 position. All the remaining reactions take place in the ER (Nagan and Zoeller, 2001; Wanders, 2014). The first reaction at the ER is catalyzed by the lysophosphatidate acyltransferases (AAG3P-AT), which links an acyl chain from acyl-CoA to the position *sn*-2 of the glycerol moiety. In the following step, phosphatidate phosphohydrolase 1 (PAP-1) removes the phosphate of 1-alkyl-2-acyl-*sn*-glycero-3-phosphate. Next, phosphoethanolamine (coming from CDP-ethanolamine) is attached to the hydroxyl group at position *sn*-3 of the glycerol moiety by the enzymatic action of ethanolamine phosphotransferase, EPT, yielding plasmanyl-PE. From that, PE-Pls is formed via oxidation catalyzed by an ER desaturase to form the vinyl double bond. Recently, the gene that encodes plasmenylethanolamine desaturase in humans has been identified as transmembrane protein 189 (TMEM189) (Werner et al., 2020).

Plasmalogen biosynthesis has been proposed to be regulated by the modulation of the rate-limiting reaction, that is, the one catalyzed by Far1. This is supported by the findings that Far1 levels (but not those of DHAP-AT, AGPS, or AADHAP-R) are elevated in plasmalogen-deficient cells (Honsho et al., 2010; Honsho and Fujiki, 2017; Kimura et al., 2018). That is, plasmalogen levels are regulated by a negative feedback mechanism. It has been shown that Far1 protein levels decrease at normal plasmalogen levels but increase significantly upon decrease in plasmalogen content (Honsho et al., 2010; Honsho and Fujiki, 2017; Kimura et al., 2018). This is not a result of a change in Far1 expression, but rather in the rate of degradation of Far1, which is increased via a mechanism dependent on posttranslational modification (Honsho et al., 2017). It has been recently proposed that the levels of Far1 are regulated by sensing the content of PE-Pls in the inner leaflet of the plasma membrane in cultured cells (Honsho et al., 2017). Bypassing the peroxisomal reactions by administration of a plasmalogen precursor, an alkylglycerol (AG), increased plasmalogen biosynthesis and induced the degradation of Far1 (Nagan et al., 1997; Nagan and Zoeller, 2001; Honsho et al., 2008, 2010, 2013; Bozelli et al., 2020b). Administration of AG to young rats shows restoration of plasmalogen levels in all tissues but the brain (Das et al., 1992). The regulation of the biosynthesis in tissues is less well characterized. While it is reasonable to propose that the regulation of plasmalogen synthesis in tissues would occur by a negative feedback mechanism, as the one reported in cultured cells, in tissues there is the possibility that the regulation might involve other mechanisms.

### Degradation

The steady state levels of plasmalogens are a result of their rate of biosynthesis and degradation. In the brain, one of the organs with highest PE-Pls content, it seems that there are two pools of PE-Pls. In white matter, PE-Pls are mainly found in the myelin sheath where its content is kept at a relatively constant level (Rosenberger et al., 2002). In gray matter, PE-Pls present a high turnover rate with a half-life of *ca.* 20 min. There are several ways that could lead to the degradation of plasmalogens, these include (i) removal of headgroup, (ii) oxidation of the enyl-ether bond, and hydrolysis of the (iii) alkyl chain and (iv) acyl chain.

Degradation of plasmalogen could occur by removal of the headgroup by a phospholipase C or D. It has been shown that PE-Pls can be the substrate of a phospholipase C, which yields 1-alkenyl-2-acyl-*sn*-glycerol (Wolfs et al., 1985). While the action of phospholipase D on PE-Pls yields 1-alkenyl-2-acyl-*sn*-phosphatidic acid (plasmenylphosphatidate) (van Iderstine et al., 1997). The enyl-ether bond can also be a site of action for plasmalogen degradation. It has been reported that the enyl-ether bond is sensitive to radical attack (by ROS and RNS) upon oxidative stress (Nagan and Zoeller, 2001; Zoeller et al., 2002). The major products of radical attack are eicosatetraenoic acid hydroxylated, 2-monoacylglycerol phospholipid, pentadecanol, formic acid, α-hydroxyaldehyde of various chain lengths, 1-formyl-2-arachidonoyl glycerophospholipid, and lysophospholipid (Gorgas et al., 2006). In addition, the enyl-ether bond can be attacked by cytochrome c upon oxidative stress leading to the formation of α-hydroxy fatty aldehydes and 2-arachidonoyl-lysophospholipid (Jenkins et al., 2018). Finally, plasmalogen can be degraded by the action of phospholipases A2 (PLA2), which cleaves the acyl chain at the *sn*-2 position yielding free fatty acid and lysoplasmalogen (Yang et al., 1996). Since plasmalogens are usually enriched with PUFA at the sn-2 position, which themselves are lipid bioactive molecules, the action of PLA2 has received special attention. There has been the identification of plasmalogen-selective PLA2, including one from rat pancreas, which is selective for AA (Hazen et al., 1990; Ford et al., 1991; Yang et al., 1996, 1997). Lysoplasmalogens may, then, be further degraded by lysoplasmalogenases or reacylated to restore plasmalogens (Arthur et al., 1986; Jurkowitz-Alexander et al., 1989; Jurkowitz-Alexander and Horrocks, 1991).

## Chronic Inflammation and Plasmalogen

**FIGURE 3:**
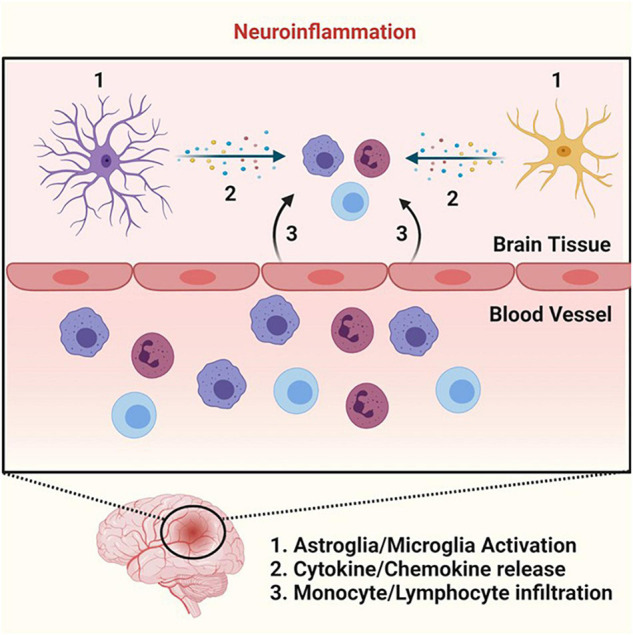
Neuroinflammation. Cartoon summarizing the key events that lead to neuroinflammation. Neuroinflammation begins with microglia and astroglia activation (step 1) that stimulates the release of various cytokines and chemokines (step 2). The cytokine and chemokine production leads to the recruitment of monocytes and lymphocytes and their subsequent infiltration into the parenchyma (step 3), allowing these immune cells to perform their necessary functions in the process of neuroinflammation. Schematic representations were generated using Biorender (©BioRender - biorender.com).

Inflammation is an important immune response that our body uses to protect itself from infection and injury (Medzhitov, 2008). Inflammation localized inside the brain and spinal cord is generally described as neuroinflammation (Figure 3) (DiSabato et al., 2016). Dysregulation of inflammation leads to chronic inflammation. Chronic inflammation has been identified as a common element in pathophysiological conditions where plasmalogens levels were reported to be decreased.

The inflammatory pathway is coordinated by complex regulatory networks that rely on signals coming from 4 distinct functional groups, those are: the (i) inducer (initiate the cascade of both cellular and molecular events), (ii) sensor (activated by the inducer), (iii) mediator (produced upon sensor activation), and (iv) effector (tissues/organs with functional states altered by the mediator to elicit the desired inflammatory response). For instance, in the case of neuroinflammation triggered by bacterial infection, receptors of the innate immune system (such as Toll-like receptors, TLRs – the sensor) on microglia and astrocytes recognize bacteria (the inducer) (Figure 3) (Medzhitov, 2008). Toll-like receptors activation leads to a coordinated cascade of events via the production of mediators, which culminates in the recruitment of leukocytes (such as monocytes and lymphocytes – the effectors) at the site of infection (Figure 3) (Zhou et al., 2006). In this process, various mediators are produced including cytokines, chemokines, and lipid mediators that will allow leukocytes to become activated to fight the pathogen at the infection site. Once the pathogens are defeated by the immune system, there is a switch from pro-inflammatory to anti-inflammatory response (the resolution phase). This simplified sequence of events depicts a successful acute inflammatory response (Medzhitov, 2008). However, if the acute inflammatory response fails, a chronic inflammatory state develops.

Lipid mediators are crucial signaling molecules involved in the inflammatory response and its resolution (the return of tissue to homeostasis). Hence, any dysregulation in the production of these lipid mediators could lead to chronic inflammation and excessive tissue damage, which, in turn, would lead to a disease state (Fullerton et al., 2014). Several lipid mediators are derived from the metabolism of PUFA such as AA, DHA, and eicosapentaenoic acid (EA, ω-3, 20:5). For instance, oxidation of AA to produce prostaglandins, thromboxanes, and leukotrienes are involved in pro-inflammatory response, while the metabolism of AA to yield lipoxins, and that of DHA and EA to yield resolvins, protectins, and maresins are involved in the anti-inflammatory response (Denisenko et al., 2020). These PUFA are essential fatty acids and need to be obtained through diet. However, they are not found in the body in their free acid form, rather esterified to glycerophospholipids. Plasmalogens are predominantly enriched with AA and DHA at the *sn*-2 position of the glycerol moiety and, therefore, they are proposed to play a role in inflammation by acting as reservoirs of these important lipid mediators. For example, up to 40% of the PE-Pls in macrophages and neutrophils (immune cells important in the inflammation process) contain AA at the *sn-2* position, which constitutes 75% of AA in these cells (Sugiura et al., 1983; Kayganich and Murphy, 1992). Upon lipopolysaccharide (LPS) stimulation (an inflammatory inducer), plasmalogens from macrophage cells are prone to hydrolysis to release AA to produce pro-inflammatory eicosanoids (Gil-de-Gómez et al., 2017).

Plasmalogens could also contribute to inflammation via modulation of membrane physical properties. For instance, plasmalogens have been shown to be critical in determining proper membrane fluidity and lipid domain formation for efficient signal transduction events (Rubio et al., 2018). In the brains of Alzheimer’s disease mice models, it has been reported that plasmalogens function by modulating TLR4 endocytosis and, consequently, decreasing the production of inflammatory cytokines, which, in turn, reduces the inflammatory phenotype (Ali et al., 2019). It was proposed that this role was due to either inhibition of clathrin-dependent endocytosis and/or enhancement of caveolin/lipid raft-mediated endocytosis (Cai et al., 2013; Ali et al., 2019). In addition, lysoplasmalogens (produced via the hydrolysis of the acyl chain at the *sn*-2 position of plasmalogens) have been proposed to actively participate in the inflammatory process via promotion of neutrophil adherence to the endothelium (White et al., 2007). PE-Pls containing AA can also act as an intermediate in the production of anandamide (arachidonoyl ethanolamine, an endocannabinoid), which has anti-inflammatory properties mediated by binding to cannabinoid receptors in the brain (Rettori et al., 2012). Anandamide can also serve as a reservoir of AA and, therefore, the production of eicosanoids (Cravatt et al., 1996). It, thus, seems reasonable that a decrease in plasmalogen levels could impair the inflammatory response by a variety of molecular mechanisms ranging from specific interactions to secondary effects on membrane physical properties.

## Lowered Plasmalogen Levels in Pathophysiological Conditions

Recently, there has been an increased attention devoted to plasmalogens. This is a consequence of the identification that in several pathophysiological conditions the levels of plasmalogens are altered. In conditions ranging from aging to degenerative and metabolic disorders it has been shown that the levels of plasmalogens are decreased. However, it is not completely understood at present the molecular mechanisms governing the decrease of plasmalogen levels in these pathophysiological conditions. In this section we will discuss the plasmalogen-related changes in some of these conditions.

### Aging

Aging in humans is accompanied by a host of molecular and cellular changes that could lead to impaired cell function and organ failure, which, in turn, could trigger degenerative processes (Giorgi et al., 2018). For instance, as one ages mitochondrial function decreases and oxidative stress increases (Boss and Seegmiller, 1981; Bektas et al., 2018; Giorgi et al., 2018; Liguori et al., 2018). In addition, in older individuals chronic inflammation develops (Ferrucci and Fabbri, 2018). Several mechanisms lead to inflammatory processes upon aging including genetic, cellular malfunction, and oxidative stress caused by defective mitochondria (Boss and Seegmiller, 1981; Bektas et al., 2018; Ferrucci and Fabbri, 2018; Giorgi et al., 2018; Liguori et al., 2018). Plasmalogen levels also change as a function of human age (Table 2). Plasmalogen content in newborns is extremely small (*ca.* 7% of the total phospholipid mass in the brain) (Farooqui et al., 2008). In the first year of life there is a dramatic increase (8-fold) in PE-Pls in brain white matter (Farooqui et al., 2008). Plasmalogen levels keep increasing linearly up to 30–40 years of age and then, at 70 years of age a significant (linear) decrease in their levels is observed (Rouser and Yamamoto, 1968). Indeed, with elderly individuals (70 years of age), it has been shown that PC-Pls and PE-Pls content in the serum (present within lipoproteins for transport from the liver to other organs) dropped 40% in comparison to healthy young controls (Maeba et al., 2007). This correlation between decreased plasmalogen levels and aging was also confirmed in studies examining mammalian tissue (Brosche and Platt, 1998). While there is no present explanation for the molecular mechanisms leading to lower plasmalogen levels upon aging, it has been noted that aging cause lower plasmalogen levels by either impairing the biosynthesis and/or increasing degradation (likely, caused by oxidative stress) (Terlecky et al., 2006; Jenkins et al., 2018).

**TABLE 2:** Plasmalogen-related abnormalities in different pathophysiological conditions.

	**Control**	**Aging**	**RCDP**	**ZS**	**AD**	**PD**	**MS**	**BTHS**	**CAD**
Plasmalogen Biosynthesis	N	N	D	D	D	N	N	N	N
Plasmalogen Degradation	N	D	N	N	D	D	D	D	D
Proposed mechanism of plasmalogen content change	-	Increased turnover Increased oxidative stress	RCDP1: Defect in PEX7 gene RCDP2: Lower activity in DHAP-AT RCDP3: Lower activity in AGPS	Absence of peroxisomes	Decreased peroxisomes in neurites Increased oxidative stress	Increased oxidative stress	Increased oxidative stress MS-related demyelination	Increased expression of iPLA2β	Increased oxidative stress
Reported plasmalogen content	-	Approximately 40% lower in elderly	Variable decrease in plasmalogen content depending on disease severity	Near absence plasmalogens	40 mol% loss in white matter 10 mol% loss in gray matter 30 mol% loss in gray matter in severe dementia	20% - 60% plasmalogen loss	General decrease in plasmalogen content	10% - 28% ethanolamine plasmalogen loss. Highest decrease seen in liver (28%) and BTHS lymphoblasts (25%)	General decrease in plasmalogen content
Plasmalogen species affected	-	Both ethanolamine and choline plasmalogens affected	Primarily ethanolamine plasmalogens affected	Both ethanolamine and choline plasmalogens near absent	Primarily ethanolamine plasmalogens affected	Primarily Ethanolamine plasmalogens affected	Both choline and ethanolamine plasmalogens affected	Choline plasmalogens affected in heart Ethanolamine plasmalogens affected in other organs	Primarily choline plasmalogens affected
Reference	-	Terlecky et al., 2006; Maeba et al., 2007; Jenkins et al., 2018	Itzkovitz et al., 2012; Dorninger et al., 2014; Noguchi et al., 2014	Itzkovitz et al., 2012; Dorninger et al., 2014; Noguchi et al., 2014	Ginsberg et al., 1995; Han et al., 2001; Igarashi et al., 2011; Sachdev et al., 2013	Fabelo et al., 2011; van Horssen et al., 2019; Mawatari et al., 2020; Patergnani et al., 2021	Fabelo et al., 2011; van Horssen et al., 2019; Mawatari et al., 2020; Patergnani et al., 2021	Kimura et al., 2018, 2019; Bargiela and Chinnery, 2019	Meikle et al., 2011; Christodoulidis et al., 2014; Sutter et al., 2016

*An outline of different pathophysiological conditions where plasmalogens levels have been reported to be decreased and the proposed effect on plasmalogen biosynthesis, degradation, and mechanism of plasmalogen content change. RCDP, Rhizomelic chondrodysplasia punctata; ZS, Zellweger’s syndrome; AD, Alzheimer’s disease; PD, Parkinson’s disease; MS, Multiple sclerosis; BTHS, Barth syndrome; CAD, Coronary artery disease.*

### Peroxisomes Diseases

Peroxisomes are organelles that play an important role in the metabolism of lipids and radical species (ROS/RNS) and, therefore, are modulators of a variety of signaling pathways dependent on them, including inflammation and immune response (Fransen et al., 2017). Peroxisomes *de novo* biogenesis emerges from a hybrid of mitochondrial and ER-derived pre-peroxisomes (Sugiura et al., 2017). Furthermore, peroxisomes act in concert with mitochondria in several metabolic processes (Sugiura et al., 2017). Therefore, it is not unexpected that dysfunction in one organelle tends to affect the other. Peroxisome diseases are the name given to the collection of pathologies caused by mutations in the genes encoding proteins involved in either peroxisomes biogenesis or function. Since, peroxisomes are the place where the *de novo* biosynthesis of plasmalogens is started, it is not surprising that in peroxisome diseases there is a decrease in plasmalogen levels. In mice models of peroxisomal deficiency diseases, neuroinflammation is an established feature, suggesting that peroxisomes play an important role against degeneration and inflammation in the brain (Kassmann et al., 2007). Below a discussion of the plasmalogen-related changes in two peroxisomal deficiency diseases will be made.

#### Zellweger’s Syndrome

Zellweger’s Syndrome (ZS) is a rare autosomal recessive disorder characterized by a defective peroxisome biogenesis, a consequence of mutations in one of the 13 PEX genes that are responsible for peroxisome formation and function (Steinberg et al., 2020). In ZS, peroxisomes are deficient, mitochondria dysfunctional, oxidative stress increased, and there is neuroinflammation (Heymans et al., 1983; Baumgart et al., 2001; Kassmann et al., 2007). In post-mortem tissue of infants with ZS, plasmalogen levels are decreased significantly in comparison to controls (Table 2) (Heymans et al., 1983, 1984). The extent of decrease varies with tissue and could be as low as 10% of that found in controls. In the brain, kidney, and liver of ZS patients PE-Pls is the plasmalogen species affected, while in muscle and heart PC-Pls is the species affected (Heymans et al., 1983, 1984).

#### Rhizomelic Chondrodysplasia Punctata

Rhizomelic Chondrodysplasia Punctata (RCDP) is a rare autosomal recessive disorder characterized by defective plasmalogen biosynthesis, a consequence of mutations in peroxisomal enzymes involved in this pathway (Table 2) (Bams-Mengerink et al., 2013). The most common type of RCDP (RCDP1) has been associated with defects in the PEX7 gene, that encodes a peroxisome import receptor responsible for the proper targeting of PTS2-proteins into peroxisomes (Itzkovitz et al., 2012). Defects in enzymatic function or expression levels of peroxisomal enzymes responsible for initiating plasmalogen synthesis, DHAP-AT and ADHAP-S, are also associated with less frequent RCDP2 and RCDP3, respectively (Itzkovitz et al., 2012; Noguchi et al., 2014). The severity of RCDP phenotype seems to correlate with plasmalogen content in fibroblasts derived from RCDP patients (Dorninger et al., 2014). For a non-severe RCDP, a 40% reduction in PE-Pls content was reported, while that value increased to more than 70% in the severe phenotype (Dorninger et al., 2014). Hence, in the case of RCDP it has been proposed that the low plasmalogen levels might be responsible for the symptoms of RCDP (Braverman et al., 2002; Itzkovitz et al., 2012; Bams-Mengerink et al., 2013; Duker et al., 2017).

### Neurodegenerative Disorders

Neurodegenerative disorders are diseases that involve the deterioration of the brain due to the progressive loss of structure and/or function of neurons, which might lead to cell death. Diseases that occur because of neurodegeneration include Alzheimer’s disease (AD), Parkinson’s disease (PD), and Multiple sclerosis (MS) (Gitler et al., 2017). Mitochondrial dysfunction and oxidative stress are key players in neurodegeneration (van Horssen et al., 2019; Patergnani et al., 2021). In neurodegeneration, mitochondrial-derived RNS/ROS can trigger inflammation as well as stimulate immune signaling cascades to intensify the inflammatory process (Heneka et al., 2014; Patergnani et al., 2021). In addition, one observation that has started to gain increased interest is the fact that in several neurodegenerative disorders a marked decrease in plasmalogen levels has been reported. This opens a new and exciting avenue of research in the field of neurodegenerative disorders. This section will expand on the relationship between plasmalogen loss and different neurodegenerative disorders.

#### Alzheimer’s Disease

Alzheimer’s Disease (AD) is a neurodegenerative disorder characterized by the presence of neurofibrillary tangles, amyloid-β plaques, synaptic loss, and abnormal Tau proteins in the brain (Ginsberg et al., 1995; Guan et al., 1999; Han et al., 2001). These molecular changes result in progressive memory loss alongside mitochondria dysfunction and oxidative and inflammatory damage to the brain (Ginsberg et al., 1995; Guan et al., 1999; Han et al., 2001; Braverman and Moser, 2012). Post-mortem analyses of the brains of AD patients have shown a decrease in PE-Pls and PC-Pls in both gray and white matter of their brains (Table 2) (Ginsberg et al., 1995; Igarashi et al., 2011). At the earlier stage of the disease, the loss of plasmalogen in AD patients is higher in white matter (40 mol%) than in gray matter (10 mol%) (Han et al., 2001). As the disease progresses to severe dementia, the gray matter plasmalogen loss increases to around 30 mol% (Han et al., 2001). Given that AD is primarily a disease of gray matter, a positive correlation between disease progression and plasmalogen loss is seen (Han et al., 2001; Sachdev et al., 2013). However, the correlation between plasmalogen loss and AD has been questioned by a recent study where it has been shown a lack of correlation between low plasmalogen levels and the ApoE4, a biomarker of AD (Han, 2005; Braverman and Moser, 2012).

#### Parkinson’s Disease

Parkinson’s Disease (PD) is a neurodegenerative disease characterized by the presence of fibrillar aggregates of α-synuclein within Lewy bodies and the associated loss of dopaminergic cells within the basal ganglia of patients, which leads to motor function impairment (Olanow et al., 2009; Miller and O’Callaghan, 2015; Powers et al., 2017; Bozelli et al., 2021). The progression of PD is associated with dysfunctional mitochondria, increased oxidative stress, and neuroinflammation (van Horssen et al., 2019; Patergnani et al., 2021). Recent literature examining PD patients has identified the presence of altered plasmalogen levels (Table 2). Although not as excessive of a decrease as in AD, ethanolamine head group-containing ether lipids decreased 30% in both plasma and erythrocytes of PD patients (Mawatari et al., 2020). It has been proposed that in PD, plasmalogen loss at lipid domains from cortical gray matter could lead to impaired cellular signaling (Fabelo et al., 2011).

#### Multiple Sclerosis

Multiple Sclerosis (MS) is a chronic neurodegenerative disease of the central nervous system (Huang et al., 2017). Believed to be an autoimmune disorder, it occurs due to infiltration of autoreactive lymphocytes across the blood brain barrier into the central nervous system (Trapp and Nave, 2008). Autoreactive lymphocyte invasion leads to localized inflammation, demyelination, axonal loss, and gliotic scarring (Trapp and Nave, 2008). In MS, mitochondrial dysfunction drives neuroinflammation, likely via an oxidative stress mechanism (Bargiela and Chinnery, 2019). While the plasmalogen-specific literature surrounding MS is inchoate, recently a marked decrease in plasmalogen (PC-Pls and PE-Pls) content in the serum of MS patients experiencing both remission and relapse of MS has been reported (Table 2) (Ferreira et al., 2021). It has been proposed that this decline in plasmalogen species in MS patients might have various causes, including (i) increased immune system stress contributing to the reduction of plasmalogen via its oxidation, and (ii) MS-related demyelination, which might also contribute to plasmalogen loss as the myelin sheath is enriched in plasmalogen species (Ferreira et al., 2021).

### Heart Diseases

Heart diseases are the first cause of death in western countries. These are a group of conditions that affect the structure and function of the heart, which could arise due to different molecular and cellular events. The heart is an organ that relies heavily on aerobic metabolism and, therefore, mitochondrial dysfunction plays a crucial role in many heart diseases (Martín-Fernández and Gredilla, 2016). Mitochondria dysfunction can increase oxidative stress, which can activate the inflammasome and lead to chronic inflammation in cardiometabolic diseases (Patergnani et al., 2021). Inflammation and oxidative stress have been proposed to play a role in the initiation, progression, and complications of cardiometabolic diseases (Tousoulis et al., 2008). Recently, the involvement of plasmalogen in heart diseases has started to emerge; specifically, the decrease in PC-Pls, which is the main plasmalogen in the heart and could constitute up to 40 mol% of the total choline phospholipids (Heymans et al., 1983; Diagne et al., 1984; Kimura et al., 2016). In this section, a discussion of plasmalogen-related changes in a couple of heart diseases will be made.

#### Barth Syndrome

Barth Syndrome (BTHS) is a rare genetic disorder, which mainly affects the heart, but also muscles, the immune system, and growth. BTHS is characterized by mutations in tafazzin, a phospholipid-lysophospholipid transacylase that is involved in the last step of the *de novo* biosynthesis of the mitochondrial-specific lipid cardiolipin (CL) (Barth et al., 1996; Bione et al., 1996; Vreken et al., 2000; Schlame et al., 2003). Barth Syndrome patients present altered content and molecular species of CL as well as abnormal mitochondrial structure and function (Vreken et al., 2000; Schlame et al., 2003; Valianpour et al., 2005; Xu et al., 2005; Gonzalvez et al., 2013; Wang et al., 2014; Goncalves et al., 2021). Moreover, a link has been reported between inflammation and mitochondria in the pathology of BTHS (Wilson et al., 2012). While alterations in CL and mitochondria have been the focus in BTHS research, lately it has been acknowledged that there are more widespread lipid changes in BTHS. A particularly important one is the observation that plasmalogen levels decreased markedly in several organs (brain, heart, and liver) of a tafazzin knockdown mouse model of the disease as well as in lymphoblast cells derived from BTHS patients (Table 2) (Kimura et al., 2018, 2019). The changes in plasmalogen are much higher than those observed for CL. In the heart, PC-Pls is the plasmalogen affected, while in brain, liver, and lymphoblasts derived from BTHS patients PE-Pls is the lipid species affected most (Kimura et al., 2018, 2019). The exact reason for this PC-Pls deficiency is unknown; however, it has been suggested that it might be related to the observed increase in the expression of iPLA_2_β (a calcium-independent phospholipase A2 that is plasmalogen-selective), in the hearts of tafazzin knockdown mice (Kimura et al., 2018).

#### Coronary Artery Disease

Coronary Artery Disease (CAD) is the most common type of heart disease. It is caused by the development of atherosclerotic plaques (lipid deposits, mainly cholesterol) inside arterial walls over time, which could end up in occlusion and, consequently, acute myocardial infarction (AMI) (Sutter et al., 2016). While lipid accumulation has been the major focus of the research on plaque formation and destabilization, more recently literature has emphasized the key roles of chronic inflammation, mitochondria dysfunction, and oxidative stress on these processes (Christodoulidis et al., 2014). Previous data has shown that lipids such as cholesterol, glycerophospholipids, sphingolipids, and triacylglycerols are important risk factors for atherogenesis (Sutter et al., 2016). Plasmalogens have also been reported to be altered in CAD (Table 2). It has been reported that PC-Pls levels are decreased in the plasma of CAD patients, specifically four molecular species of PC-Pls (33:1, 33:2, 33:3, and 35:3) were reduced (Meikle et al., 2011; Sutter et al., 2016).

As illustrated by the discussion above, the recent interest in plasmalogens by the scientific community is not surprising. While the relationship between plasmalogen loss and these various pathophysiological conditions is clear, there is a lack of understanding of the molecular mechanisms. However, in some of these conditions a common scenario has started to emerge; that is, mitochondria dysfunction triggers oxidative stress, which, in turn, leads to depleted plasmalogens and chronic inflammation. Hence, it seems reasonable to propose that a decrease in plasmalogen levels is tightly linked with these biological processes. The literature has established general roles that plasmalogens play within the cellular environment; however, more in-depth analysis of these functions is necessary as well as the dependence on their molecular species (Dean and Lodhi, 2018). This data could provide a means of diagnosis, prognosis, and/or treatment.

## Restoring Plasmalogen Levels as a Therapeutic Strategy

In conditions with altered lipid metabolism, a therapeutic strategy that has been considered involves the use of small molecules that could restore lipid homeostasis. Hence, the observation that plasmalogen levels are decreased in several pathophysiological conditions opens a new avenue for the development of potential therapies to these conditions, that is, plasmalogen replacement therapy (PRT). The idea behind PRT is to administer purified plasmalogens and/or plasmalogen precursors to normalize plasmalogen levels.

Administration of plasmalogen and/or its precursors has been utilized in different clinical settings to increase plasmalogen levels as well as a strategy to prevent/attenuate different pathological conditions (Das et al., 1992; Marigny et al., 2002; Brites et al., 2011; Bozelli et al., 2020b). One of the most used small molecules in PRT is AG, which is a plasmalogen precursors that enters the biosynthesis pathway in the ER after being phosphorylated in the cytosol (Synder, 1992; Braverman and Moser, 2012). For instance, AG has been shown to restore plasmalogen levels in fibroblast cells derived from ZS and RCDP patients (Brites et al., 2004). Administration of AG to a cell model of BTHS restored plasmalogen level and partly CL levels as well as improved mitochondria fitness (Bozelli et al., 2020b). In a mouse model of RCDP (*Pex7*^hypo/null^) ingestion of a synthetic vinyl-ether plasmalogen restored plasmalogen levels in the plasma and increased the content at different extents in other tissues (with exception of the brain, lung, and kidney) (Fallatah et al., 2020). In addition, the treatment normalized the hyperactive behavior of *Pex7*^hypo/null^ (Fallatah et al., 2020). In the brain of the Pex7 knockout mice, AG diet also did not rescue plasmalogen levels (Brites et al., 2011). However, DHA-enriched lipids have been shown to increase PE-Pls levels in the brain and, consequently, ameliorate the phenotype in a dementia mice model (Zhao et al., 2020). In a rat model of AD, ingestion of purified PE-Pls derived from *Ascidia viscera* improved cognition and learning ability (Yamashita et al., 2017). In mice models of PD, ingestion of plasmalogens or their precursors led to improved neuroprotection and immunomodulation as well as reduced neuroinflammation (Hossain et al., 2018; Nadeau et al., 2019). In ZS, PRT has been used in two patients where it has been shown to increase the levels of plasmalogen upon AG ingestion (Wilson et al., 1986). In addition, it also has been reported that memory function of AD patients with mild symptoms can be improved upon ingestion of scallop-derived plasmalogens (Fujino et al., 2017). In PD patients, ingestion of scallop-derived plasmalogens increased blood plasmalogen concentration as well as improved non-motor symptoms of PD (Mawatari et al., 2020).

The reported changes in plasmalogen levels in several diseases where chronic inflammation plays a key role opens a new avenue for their treatment. Contrary to the manipulation of the levels of other phospholipids, restoring plasmalogen levels via the use of PRT has been shown to be very successful in several disease models studied. It is crucial to understand both how plasmalogen levels are decreased as well as how their levels could be restored at the molecular level for the design of better, more potent, small molecules in clinical applications of PRT.

## Lowering Plasmalogens in Disease: Cause or Effect?

The steady-levels of plasmalogens are determined by their rate of biosynthesis and degradation. Alterations in plasmalogen metabolism and/or catabolism are, therefore, associated with changes in their levels. While this is a reasonable generic explanation for the alteration in plasmalogen content, the exact molecular mechanism varies with the pathophysiological condition. For instance, in peroxisome diseases plasmalogen content loss is, usually, a result of impaired biosynthesis. In ZS plasmalogen biosynthesis is deficient due to a lack of functional peroxisomes, while in RCDP the impaired biosynthesis is a consequence of mislocalization and/or absence of functional peroxisomal enzymes responsible to initiate plasmalogen biosynthesis, such as DHAP-AT and AGPS (Nagan and Zoeller, 2001; Brites et al., 2004). On the other hand, during aging as well as in degenerative (AD, PD, MS) and metabolic (BTHS, CAD) diseases, it seems that plasmalogen degradation enhancement is responsible for the lowering in plasmalogen levels. In all these conditions, mitochondria are dysfunctional, and there is an increase in the inflammatory response and oxidative stress. One way to degrade plasmalogens is via the oxidation of the enyl-ether bond, a condition that is favored upon increasing oxidative stress in the cell (Hahnel et al., 1999b; Zoeller et al., 2002). In addition, it has been reported that there is a link between oxidative stress and the activity of enzymes along the plasmalogen degradation pathways, such as cytochrome c (which acts as a plasmalogenase cleaving the enyl-ether bond) and cytosolic PLA2 (which hydrolyzes AA at the *sn*-2 position to produce eicosanoids) (Chuang et al., 2015; Jenkins et al., 2018; Kimura et al., 2018).

There is a good correlation between diseases with chronic inflammation and a lower level of plasmalogens (Wang, 1999; Spiteller, 2006). Conversely, administration of plasmalogens to individuals with these diseases reduces the extent of inflammation. Inflammation is a factor in aging, and it has been shown to play a key role in degenerative and metabolic diseases. One of the best studied examples is AD. It has been shown in postmortem brains that there is a 60% decrease in PE-Pls relative to PE in affected brain regions of AD patients. Furthermore, this decrease in PE-Pls was specific to brain regions with histological damage characteristic of the disease and not in unaffected regions of the brain of the same individual (Ginsberg et al., 1995). This lower level of PE-Pls is, however, not limited to regions of the brain where there is morphological damage but is even seen in the levels of PE-Pls in circulation, which correlated with a characteristic AD biomarker, *i.e.*, an increased level of the protein Tau in the cerebrospinal fluid (Kling et al., 2020). The role of plasmalogens in AD has been recently reviewed (Su et al., 2019). Inflammation caused by the administration of bacterial LPS resulted in a decreased level of plasmalogen in the brain as well as the accumulation of Aβ peptides. These changes were reversed by the administration of plasmalogens (Ifuku et al., 2012). In addition to AD, other neurodegenerative diseases are associated with aging that results in the decline of plasmalogen levels caused by defects in the ability of peroxisomes to synthesize plasmalogens (Jo and Cho, 2019). Peroxisomes also contribute to the production of cytokines during inflammation (di Cara et al., 2017). Peroxisomal lipid synthesis regulates inflammation by sustaining neutrophil membrane phospholipid composition and viability (Lodhi et al., 2015). Peroxisomal alterations in the brains of patients with AD and with PD suggest that peroxisomal defects may facilitate the development of neurodegenerative disorders (Cipolla and Lodhi, 2017; Deori et al., 2018). Neurodegenerative diseases are strongly associated with oxidative stress (Wanders, 2014; Cipolla and Lodhi, 2017). Several reviews have appeared associating oxidative stress with neurodegenerative diseases (Jiang et al., 2016; Kamat et al., 2016; Puspita et al., 2017). Neuroinflammation results in the accumulation of 2-chlorohexadecane in brain lipids of endotoxin-treated mice indicating that inflammatory conditions may deplete plasmalogen levels (Üllen et al., 2010).

The series of events that results in lowering plasmalogen levels in the brain is believed to be associated with an oxidation process (Senanayake and Goodenowe, 2019). Damaged peroxisomal functions as well as higher levels of H_2_O_2_ potentially cause permanent plasmalogen deficiency that led to membrane changes, signaling abnormalities, neurotransmission deficits, and lowering antioxidant defenses (Braverman and Moser, 2012). Oxidative stress associated with inflammation can accelerate plasmalogen degradation by cleaving the vinyl-ether bond, further reducing the anti-inflammatory and antioxidative capacity of the tissues initiating an irrevocable vicious cycle that progresses to pathological abnormalities (Su et al., 2019). It has been proposed that cytochrome c-mediated degradation of plasmalogens due to increased oxidative stress as a potential mechanism responsible for the decrease in plasmalogens (Jenkins et al., 2018). Thus, relating oxidative stress with the loss of plasmalogens leading to disease (Jenkins et al., 2018).

Elevated levels of plasmalogen peroxides relative to plasmalogens can be detected in aging brains and in AD-affected brains providing further evidence of the significance of the maintenance of plasmalogens in the intact state of the brain (Weisser et al., 1997). Inflammation has been shown to be related to apoptosis and the generation of inflammatory caspases (Davies et al., 2021). Caspases are initiators of apoptosis and neurodegeneration (Budihardjo et al., 1999). Caspases are also associated with inflammation (Wu et al., 2009). TNFα (tumor necrosis factor α), a signaling molecule produced in inflammatory conditions, induces caspase-dependent inflammation in renal endothelial cells through a Rho- and myosin light chain kinase-dependent mechanisms. Among different caspases, caspase-3 is of particular interest because it is found to be associated with the pathologies of neurodegenerative diseases, such as AD (Su et al., 2001; D’Amelio et al., 2011). Recent studies also reported that caspase-3 is associated with the formation of amyloid-β (Aβ) by processing of amyloid precursor protein (Stone et al., 2002). Caspase-8 and caspase-3 have been implicated in microglial activation by regulating protein kinase C (Burguillos et al., 2011). Plasmalogens inhibit LPS-induced Aβ formation and microglial activation in the mouse brain cortex (Ifuku et al., 2012). Plasmalogens also suppress apoptosis in intestinal tract cells by attenuating induced inflammatory stress (Nguma et al., 2021b). Dietary PE-Pls has been shown to reduce intestinal inflammation, oxidative stress, and the expression of apoptosis-related proteins in the colon mucosa (Nguma et al., 2021a). Inflammation has also been suggested to play a role in cancer (Lan et al., 2021).

In cancer and degenerative diseases, ferroptosis (an iron-dependent, non-apoptotic cell death process) plays an important role (Stockwell et al., 2017). The increase in the levels of peroxidized intracellular lipids due to the oxidation of PUFA moieties in membrane phospholipids is responsible for triggering ferroptosis (Conrad and Pratt, 2019). Recently, it has been shown that plasmalogens, which are enriched in PUFA, can induce ferroptosis by providing PUFA for lipid peroxidation (Zou et al., 2020). Likewise, plasmalogen biosynthesis has been reported to mediate a new axis of ferroptosis, which is dependent on long-chain saturated fatty acids (Cui et al., 2021). It has been shown that the enzymes Far1 and TMEM189, which catalyze reactions in plasmalogen biosynthesis (see above), can mediate the new axis of ferroptosis (Cui et al., 2021). Both ferroptosis and plasmalogens play a role in inflammatory processes (Braverman and Moser, 2012; Sun et al., 2020). However, it is not currently understood the molecular mechanisms of the interplay between ferroptosis and plasmalogens in inflammatory processes. Future research in the field will help expand our understanding of the role of plasmalogens in inflammation.

Phagocytosis by macrophages plays an important role in controlling inflammation. Brain inflammation may be a consequence of attack by macrophages (Xiong et al., 2016). Cells deficient in PE-Pls have a reduced ability to phagocytize opsonized zymosan particles (Rubio et al., 2018). This defect can be reversed by incubating the plasmalogen deficient cells with lysoplasmalogen, which, presumably, acts as a metabolic precursor to plasmalogens. Because of the increased level of plasmalogens the number and size of lipid domains in the membrane is increased, membrane fluidity is lowered to levels found in cells containing normal plasmalogen levels, and receptor-mediated signaling becomes more efficient.

Activation of protein kinase C delta (PKCδ) is linked to neuroinflammation. Knocking out PKCδ in mice results in resistance to inflammation, while upregulation of PKCδ in microglial cells promotes neuroinflammation (Ren et al., 2014; Gordon et al., 2016). Microglial activation is a pathological feature of many neurodegenerative diseases (Ali et al., 2019). The presence of activated microglia and reactive astrocytes that produce cytokines are associated with AD pathologies (Apelt and Schliebs, 2001; Tahara et al., 2006; Salminen et al., 2008). Plasmalogens have been shown to inhibit neuronal cell death by suppressing an intrinsic apoptotic pathway, which is characterized by the activation of caspase-9 (Hossain et al., 2013). It was also found that the systemic LPS-induced activation of microglial cells and the expression of pro-inflammatory cytokines were significantly attenuated by the administration of plasmalogens (Ifuku et al., 2012).

Toll-like receptors (TLR) plays a wide role in innate and adaptive immune responses upon stimulation by exogenous and endogenous TLR ligands. Among TLR, the TLR4 has attracted increased attention due to its ability to recruit different adaptor proteins. LPS-induced inflammatory signaling is associated with the endocytosis of TLR4. The pretreatment of cells with plasmalogens attenuated the LPS-induced signaling by inhibiting the dynamin-dependent internalization of TLR4. Knockdown of the plasmalogen synthesizing enzyme, DHAP-AT, by lentiviral vectors encoding short hairpin-RNA against DHAP-AT resulted in the increased activation of caspases and the endocytosis of TLR4, which was reversed by the ingestion of plasmalogens (Ali et al., 2019). The LPS-TLR4 complex initiates the TLR4 endocytosis, which is believed to play a major role in regulating inflammatory signals to induce cytokine expression by activating the Toll/interleukin-1 receptor domain-containing adaptor protein and the MyD88 adaptor proteins, as well as Toll/IL-1R domain-containing adaptor inducing type I interferons-mediated pathways in mouse macrophages and Ba/F3 cells (Akira and Takeda, 2004; Kagan et al., 2008; Wong et al., 2009). The internalization of TLR4 has been reported to be mediated by clathrin-dependent endocytosis in HEK 293 cells, lipid domain-mediated endocytosis in CHO cells, and both clathrin-dependent and lipid domain-mediated endocytosis in cortical astrocytes (Shuto et al., 2005; Husebye et al., 2006; Pascual-Lucas et al., 2014).

Inflammation is accompanied by many changes as outlined above. These include mitochondria dysfunction, oxidative stress, apoptosis and the increased expression of caspases, phagocytosis by macrophages, activation of PKCδ, stimulation of microglia with the generation of inflammatory cytokines, and TLR signaling. Many of these processes are associated with pathologies caused by inflammation and are reversed by administering plasmalogens and/or plasmalogen precursors. Plasmalogen levels are reduced in diseases that affect these processes. More complicated, however, is determining which of the processes cause the dysfunctions and which are consequences of the inflammatory process itself. It can be concluded that plasmalogens are at least protective against cell or tissue damage caused by inflammation. This is supported by the finding that in many examples of pathologies caused by inflammation, there is a loss of plasmalogens. Furthermore, administration of plasmalogens or plasmalogen precursors can prevent tissue damage caused by inflammation. A mechanism can be proposed to explain the protective effect of plasmalogens. Inflammation is often accompanied by the production of ROS causing oxidative damage to tissues. Plasmalogens are protective against oxidative damage because of their enyl-ether linkage that is highly susceptible to oxidation by ROS, thus preventing ROS from attacking at other sites. Deciding if the loss of plasmalogens is a cause or a consequence is to some extent a matter of definitions and will vary from one disease to another. In some cases, the loss of plasmalogens is the primary defect, such as inflammation related to aging in which the ability to synthesize plasmalogens is decreased. However, in other cases, such as the production of inflammatory cytokines, other processes may occur first. Nevertheless, even in these cases, plasmalogens play a protective role and can prevent inflammation. In that sense, even when the loss of plasmalogens is not the first event, their presence or absence can determine the course of inflammation.

## Author Contributions

All authors contributed to designing the focus of the review, writing sections, and editing the final manuscript.

## Conflict of Interest

The authors declare that the research was conducted in the absence of any commercial or financial relationships that could be construed as a potential conflict of interest.

## Publisher’s Note

All claims expressed in this article are solely those of the authors and do not necessarily represent those of their affiliated organizations, or those of the publisher, the editors and the reviewers. Any product that may be evaluated in this article, or claim that may be made by its manufacturer, is not guaranteed or endorsed by the publisher.

## Abbreviations

A β: Beta amyloid peptides
AA: Arachidonic acid
AD: Alzheimer’s disease
AGPS: Alkyl-dihydroxyacetone phosphate synthase
AG: Alkylglycerol
AMI: Acute myocardial infarction
BTHS: Barth Syndrome
CAD: Coronary artery disease
CL: Cardiolipin
DHA: Docosahexaenoic acid
DHAP: Dihydroxyacetone phosphate
DHAP-AT: Dihydroxyacetone phosphate acyltransferase
IL-1R: Interleukin-1 receptor
LPS: Lipopolysaccharides
MS: Multiple sclerosis
PC: Phosphatidylcholine
PC-Pls: Plasmenylcholine
PD: Parkinson’s Disease
PE: Phosphatidylethanolamine
PE-Pls: Plasmenylethanolamine
PKC δ: Protein kinase C delta
PLA2: Phospholipase A2
PRT: Plasmalogen replacement therapy
PUFA: Polyunsaturated fatty acids
RCDP: Rhizomelic chondrodysplasia punctata
ROS: Reactive oxygen species
RNS: Reactive nitrogen species
TLR: Toll-like receptors
TNF α: Tumor necrosis factor alpha
ZS: Zellweger’s syndrome.
